# Association between *ABCA1* Gene Polymorphisms and Plasma Lipid Concentration: A Systematic Review and Meta-Analysis

**DOI:** 10.3390/jpm11090883

**Published:** 2021-09-03

**Authors:** Sun-Young Shim, Ha-Young Yoon, Jeong Yee, Ji-Min Han, Hye-Sun Gwak

**Affiliations:** 1 College of Pharmacy and Graduate School of Pharmaceutical Sciences, Ewha Womans University, Seoul 03760, Korea; jimmyjam9620@gmail.com (S.-Y.S.); hayoungdymphnayoon@gmail.com (H.-Y.Y.); jjjhello1@naver.com (J.Y.); 2 Graduate School of Clinical Biohealth, Ewha Womans University, Seoul 03760, Korea; 3 College of Pharmacy, Chungbuk National University, Cheongju-si 28160, Chungcheongbuk-do, Korea

**Keywords:** *ABCA1*, 69C>T, 825V>I, 230R>C, lipid

## Abstract

Background: Although *ABCA1* gene polymorphisms may be associated with the plasma lipid concentration, the literature has not shown a consistent pattern. In this study, we attempted to elucidate the association between the *ABCA1* 69C>T, 825V>I, and 230R>C polymorphisms and the plasma lipid concentration through a systematic review and meta-analysis. Methods: We selected studies published up to October 2020 in the PubMed, Web of Science, and Embase databases according to inclusion and exclusion criteria. The mean difference (MD) and 95% confidence interval (CI) were used to assess the relationship between the presence of *ABCA1* 69C>T, 825V>I, and 230R>C and plasma lipid levels. Meta-analysis was performed using Review Manager (version 5.3). Both Begg’s test and Egger’s regression test of the funnel plot were performed using R Studio software (version 3.6.0) to identify publication bias. Results: We analyzed the data on the *ABCA1* 69C>T polymorphism involving 14,843 subjects in 11 studies, 825V>I polymorphism involving 2580 subjects in 5 studies, and 230R>C polymorphism involving 4834 subjects in 4 studies. The T allele carriers in 69C>T, II carriers in 825V>I, and C carriers in 230R>C had lower high-density lipoprotein cholesterol levels; the MD (95% CI) was −0.05 mmol/L (95% CI: −0.09 to −0.01, *p* = 0.02), −0.05 mmol/L (95% CI: −0.09 to −0.00, *p* = 0.03), and −0.1 mmol/mL (95% CI: −0.12 to −0.07 mmol/L, *p* < 0.00001), respectively. In the case of 230R>C, the serum total cholesterol concentration of C carriers was significantly lower than that of RR carriers (−0.2 mmol/L, 95% CI: −0.3 to −0.11, *p* < 0.0001). Conclusion: This meta-analysis demonstrates that the *ABCA1* 69C>T, 825V>I, and 230R>C polymorphisms could affect the plasma lipid concentration. As the plasma lipid concentration may be related to various diseases, *ABCA1* genotyping could be useful for the management of lipid levels.

## 1. Introduction

Coronary heart disease (CHD) is a common disease with high morbidity and mortality [1]. Although multiple factors affect the development of CHD, dyslipidemia is known to be one of the most important factors for CHD development [2]. Dyslipidemia refers to a condition in which total cholesterol (TC), triglyceride (TG), and low-density lipoprotein cholesterol (LDL-C) levels are increased or high-density lipoprotein cholesterol (HDL-C) levels are decreased due to abnormal lipoprotein metabolism.

ATP-binding cassette transporter A1 (ABCA1) is a cell membrane transport protein composed of 2261 amino acids. One of its main functions is to promote the efflux of cellular cholesterol and phospholipids [3]. They combine with extracellular apolipoproteins, forming incipient HDL-C. This is the first step of the reverse cholesterol transport process which transports and removes cholesterol and phospholipids from peripheral cells back to the liver, through which ABCA1 is involved in cholesterol homeostasis [4,5,6]. ABCA1 is also known to affect the plasma concentration of HDL-C [7]. A previous observational study showed that a 50% increase in ABCA1-mediated cholesterol efflux caused a 30% increase in the concentration of HDL-C, thus resulting in a decreased incidence of coronary artery disease (CAD) by 50% [8].

The *ABCA1* gene is a highly polymorphic gene located on chromosome 9 [9]. Since polymorphism refers to variations of a particular DNA sequence, a polymorphic gene can result in the production of an abnormal protein by altering the amino sequence; the variant protein may lead to its abnormal function or a change in its activity [10,11]. Some mutations of *ABCA1* have been found in the intron, exon, or promoter region, which can affect the function of ABCA1. Defects in the reverse cholesterol transport process caused by *ABCA1* genetic mutations can cause Tangier’s disease or familial HDL deficiency by reducing HDL-C levels [12].

Several studies have demonstrated the relationship between *ABCA1* genetic polymorphisms and the plasma lipid concentration, including *ABCA1* 69C>T, 825V>I, and 230R>C [9,13,14,15]. Although most mutations are located in introns, *ABCA1* 69C>T is located in the 5ʹ untranslated region, and 825V>I and 230R>C are missense mutations; thus, they may have a greater effect on the expression and function of ABCA1. However, previously published studies on the relationship between these *ABCA1* gene polymorphisms and the plasma lipid concentration showed inconsistent results.

Therefore, the aim of this study was to investigate the correlation between the plasma lipid concentration and the *ABCA1* 69C>T, 825V>I, and 230R>C polymorphisms through a systematic review and meta-analysis of previous studies.

## 2. Methods and Materials

### 2.1. Literature Search Strategy and Inclusion Criteria

This meta-analysis was conducted according to the checklist outlined in the Preferred Reporting Items for Systematic Reviews and Meta-Analyses (PRISMA) [16]. Two reviewers independently searched for studies published up to 16 October 2020. An extensive search of electronic databases (PubMed, Web of Science, and Embase) was performed using the following search terms: {ABCA1 OR (ATP-binding cassette transporter 1) OR (ATP-binding cassette transporter A1) OR (adenosine triphosphate-binding cassette transporter A1) OR (ATP Binding Cassette Sub Family A Member 1) OR (ATP Binding Cassette Transporter, Subfamily A)} AND (polymorph* OR variant* OR mutation* OR genotyp* OR allele* OR SNP*) AND {Dyslipidemia OR dyslipidemias OR HDL OR (high density lipoprotein) OR LDL OR (low density lipoprotein) OR triglyceride}.

Studies were included if they evaluated the relationship of *ABCA1* 69C>T, 825V>I, and 230R>C with the lipid levels (mmol/L) of adults. The language of the articles was limited to English. Studies were excluded if (i) they were editorials, notes, abstracts, reviews, comments, letters, news, or editorials; (ii) they were in vitro or in vivo studies; (iii) extraction of the data was not possible; or (iv) sample size was less than 20. If there were overlapping data, only the most recent and comprehensive data were included in the meta-analysis.

### 2.2. Data Extraction and Study Quality Assessment

Two reviewers extracted the data independently, and discrepancies were resolved by consensus. Extracted data included the following information: name of the first author, publication year, country, disease, study design, number of subjects, mean age, percentage of females, genotyping method, studied lipid types, and lipid levels by genotype.

Two researchers independently assessed the selected studies based on the Newcastle–Ottawa scale (NOS) for cohort studies and case–control studies [17]. The NOS has three categories: selection of study sample, comparability between the case and control groups, and outcome or exposure assessment. Each study can be assessed with a total score of 0–9. In this review, we rated 1 point for each item of comparability, if age and other known risk factors (such as body mass index (BMI)) were matched or adjusted for in the analysis.

### 2.3. Statistical Analysis

Meta-analysis was performed using Review Manager (version 5.3; The Cochrane Collaboration, Copenhagen, Denmark). The mean difference (MD) and 95% confidence interval (CI) were used to assess the relationship between the presence of *ABCA1* 69C>T, 825V>I, and 230R>C and lipid levels. For studies reporting only medians and interquartile ranges, we retrieved the mean and variance values from the authors of the original reports or used appropriate formulas to calculate the mean and variance, making no assumption on the distribution of the underlying data [18]. The unit “mmol/L” was used for all lipid variables in the meta-analysis, and unit conversion was conducted for articles in which other units were used.

The heterogeneity across studies was estimated by the chi-square test and *I*^2^ statistic. An *I*^2^ value of ≥50% was considered to indicate significant heterogeneity. The selection of the proper effect model was based on the analysis results; the fixed effect model was used if *I*^2^ < 50%, and the random effect model was used if *I*^2^ ≥ 50% [19]. Both Begg’s test and Egger’s regression test of the funnel plot were performed using R Studio software (version 3.6.0; R Foundation for Statistical Computing, Vienna, Austria) to identify publication bias [20,21]. A *p*-value < 0.05 was considered statistically significant.

## 3. Results

### 3.1. Characteristics of the Eligible Studies

**Figure 1:**
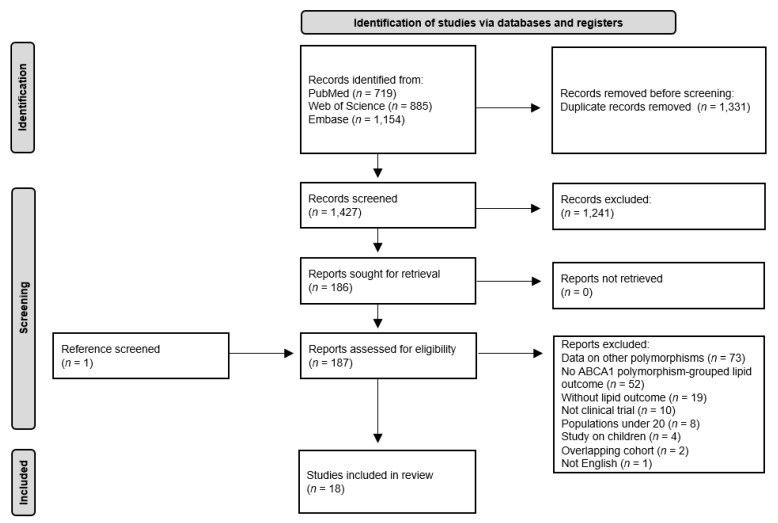
Flow diagram of study inclusion.

A detailed flow chart of the study selection process is presented in Figure 1. A total of 2758 studies were identified from searching three databases. After the removal of 1331 duplicates, 1427 records were initially identified, and the titles and abstracts were screened based on the inclusion criteria of the study. From this initial review, 186 studies were selected for full-text review and assessed for eligibility. Following manual screening, one article was added. Of these studies, 169 studies were excluded for the following reasons: data were on other polymorphisms (*n* = 73); there was no *ABCA1* polymorphism-grouped lipid outcome (*n* = 52); there was no lipid outcome (*n* = 19); the study was not a clinical trial (*n* = 10); the sample size was less than 20 (*n* = 8); the study involved children (*n* = 4); there was an overlapping cohort (*n* = 2); the study was not in English (*n* = 1). Therefore, 18 articles were identified for this systematic review [13,14,15,22,23,24,25,26,27,28,29,30,31,32,33,34,35].

The characteristics of the included studies are presented in Table 1. The studies were published from 2005 to 2020. There were eight cohort studies, nine case–control studies, and one cross-sectional study; the NOS score was between 5 and 7.

### 3.2. Association of the ABCA1 Gene with Plasma Lipid Concentration

**Figure 2:**
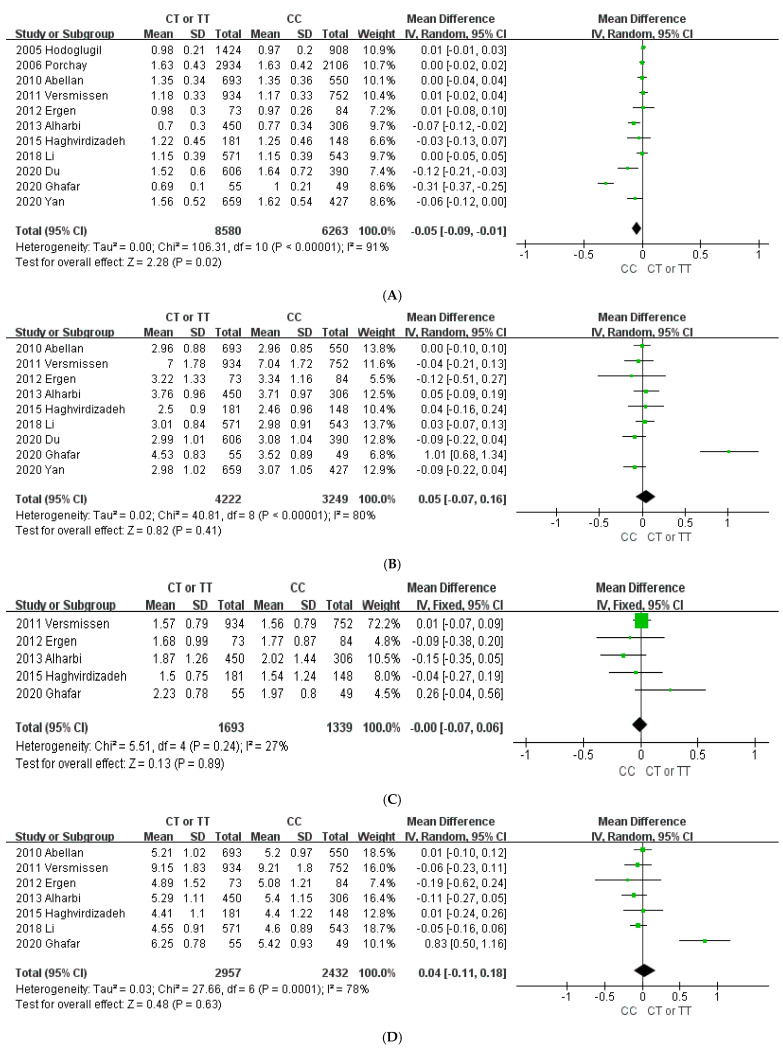
Forest plots demonstrating the association between the *ABCA1* 69C>T polymorphism and lipid level (mmol/L). (**A**) High-density lipoprotein cholesterol; (**B**) low-density lipoprotein cholesterol; (**C**) triglycerides; (**D**) total cholesterol.

To evaluate the association of *ABCA1* 69C>T with the plasma lipid concentration, 11 studies with a total of 14,843 subjects were included in the meta-analysis. As shown in Figure 2A, T allele carriers (CT+TT) had a lower HDL-C concentration of 0.05 mmol/L (95% CI: −0.09 to −0.01, *p* = 0.02) compared with the concentration of wild-type homozygote carriers (CC). High heterogeneity was detected among the studies (*I*^2^ = 91%; *p* < 0.00001). There were no significant differences in LDL-C, TG, and TC levels between genotypes (Figure 2B, Figure 2C, and Figure 2D, respectively). Neither Begg’s test nor Egger’s test showed significant publication bias in all lipid levels except for HDL-C (*p* = 0.024 and *p* = 0.071 for Begg’s test and Egger’s test, respectively; Supplementary Figure S1).

**Figure 3:**
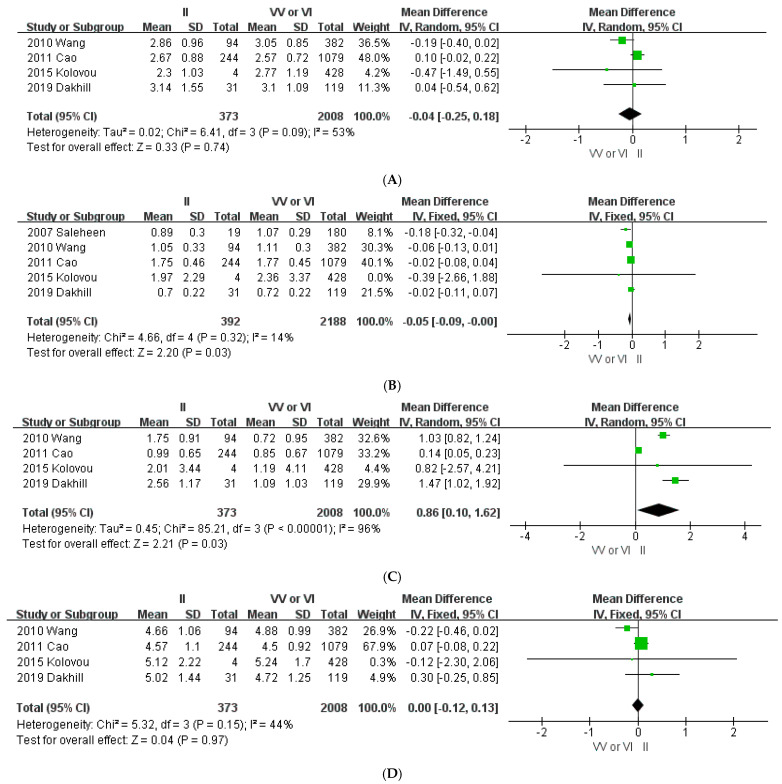
Forest plots demonstrating the association between the *ABCA1* 825V>I polymorphism and lipid level (mmol/L). (**A**) High-density lipoprotein cholesterol; (**B**) low-density lipoprotein cholesterol; (**C**) triglycerides; (**D**) total cholesterol.

To evaluate the association of *ABCA1* 825V>I with the plasma lipid concentration, 5 studies with a total of 2580 subjects were included in the meta-analysis. As shown in Figure 3A, subjects with the II genotype had a lower HDL-C concentration of 0.05 mmol/L (95% CI: −0.09 to −0.00, *p* = 0.03) compared with the concentration of subjects with the VI and VV genotypes based on a meta-analysis of five studies. Low heterogeneity was observed (*I*^2^ = 14%; *p* = 0.32). The association between the 825V>I polymorphism and plasma LDL-C, TG, and TC levels was not statistically significant (Figure 3B, Figure 3C, and Figure 3D, respectively). No publication bias was detected when using Begg’s test and Egger’s test in all lipid levels (Supplementary Figure S2).

**Figure 4:**
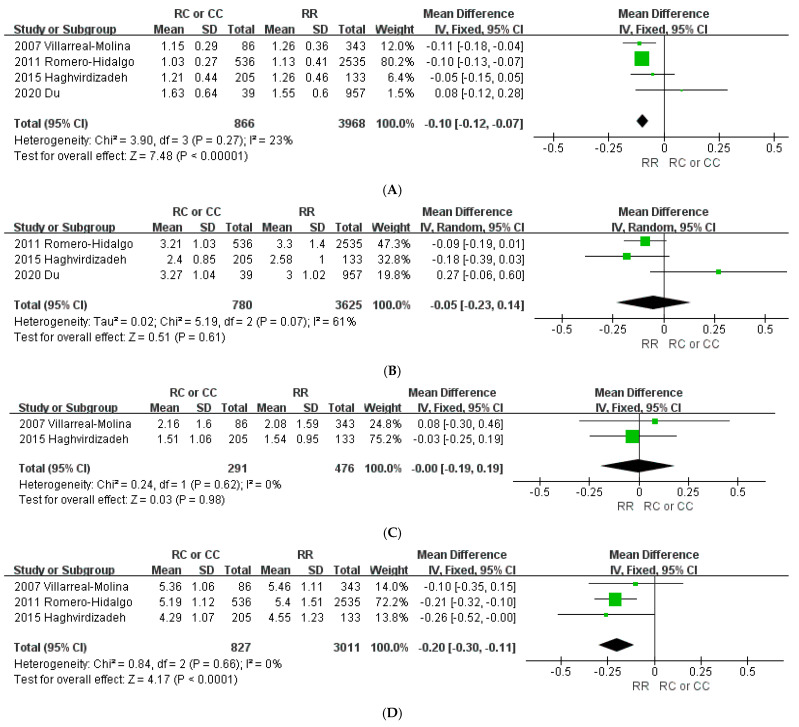
Forest plots demonstrating the association between the *ABCA1* 230R>C polymorphism and lipid level (mmol/L). (**A**) High-density lipoprotein cholesterol; (**B**) low-density lipoprotein cholesterol; (**C**) triglycerides; (**D**) total cholesterol.

To evaluate the association of *ABCA1* 230R>C with the plasma lipid concentration, 4 studies with a total of 4834 subjects were included in the meta-analysis. As shown in Figure 4A, variant C carriers had a lower HDL-C concentration of 0.1 mmol/L (95% CI: −0.12 to −0.07, *p* < 0.00001) compared with the concentration of non-carriers. Low heterogeneity was observed among the studies (*I*^2^ = 23%; *p* = 0.27). The TC concentration of variant C carriers was significantly lower than that of wild-type homozygote carriers (RR) by 0.2 mmol/L (95% CI: −0.3 to −0.11, *p* < 0.0001) (Figure 4D). No heterogeneity was observed among the studies (*I*^2^ = 0%; *p* = 0.66). There were no differences in LDL-C and TG levels between genotypes (Figure 4B and Figure 4C, respectively). Publication bias was not detected when using Begg’s test and Egger’s test except for HDL-C (*p* = 0.0415 and *p* = 0.204 for Begg’s test and Egger’s test, respectively; Supplementary Figure S3).

## 4. Discussion

This meta-analysis showed that the T allele carriers in 69C>T, II carriers in 825V>I, and C carriers in 230R>C had significantly lower HDL-C levels. In the 230R>C polymorphism, TC levels were significantly lower among C carriers compared with wild-type homozygote carriers.

ABCA1 transfers excess intracellular cholesterol and phospholipids to apolipoprotein AI, producing an early form of HDL-C [5]. This initial form of HDL-C exhibits cardiovascular protective effects by mediating a reverse transport process that exchanges cholesterol for TGs, followed by transport to the liver [6,36,37]. A prospective observational study reported that an increase of 1 mg/dL (0.026 mmol/L) in HDL-C levels reduced the risk of CHD by 2–3% [38].

The results of our meta-analysis are consistent with those of previous studies on the risk of developing lipid-related diseases such as atherosclerosis (AS) and CAD, in which abnormal lipid levels are the main cause of the diseases. The relationship between the 69C>T polymorphism and AS was examined in a previous study [39]. This meta-analysis involving a sample size of approximately 20,000 subjects reported an increased risk of AS for mutant T carriers (CT+TT) compared with wild-type homozygote carriers; however, this study did not directly compare gene polymorphisms with plasma lipid levels. Nevertheless, considering that abnormalities in plasma lipid levels are the main cause of AS, their result is consistent with ours.

In the case of the 825V>I polymorphism, the aforementioned study failed to show a significant association with the development of AS; this is different from the results of our study, in which the level of HDL-C was significantly lower in II genotype carriers than in VI and VV genotype carriers [39]. However, subgroup analysis, which was performed on subjects with CAD only, showed a significant correlation; this suggests that although *ABCA1* gene polymorphisms may play different roles in different atherosclerotic diseases, they can be related to the onset of AS.

Analysis of *ABCA1* 230R>C revealed that C carriers had both lower HDL-C and lower TC plasma levels compared with the levels of non-carriers in our study, which were controversial in explaining the association of *ABCA1* polymorphisms and the development of lipid-related diseases such as AS and CAD. Similar results were also reported in a previous study conducted in Mexico on the association between the 230R>C polymorphism and early CAD [40]. Participants with variant C showed a lower risk of developing CAD and had lower HDL-C and TC levels. This inconsistency may be attributed to the various functions of ABCA1 in each cell [5]. As ABCA1 is involved not only in cholesterol export but also in inflammation and platelet function, the *ABCA1* 230R>C polymorphism may also affect other functions, thus contributing to the inconsistency between lipid levels and the risk of developing lipid-related diseases [41,42].

This study is the first meta-analysis to reveal an association between *ABCA1* gene polymorphisms (69C>T, 825V>I, and 230R>C) and abnormal lipid levels. However, this study has several limitations. First, this study did not consider that there may be differences in the plasma lipid concentration of patients with underlying diseases such as familial hypercholesterolemia or diabetes mellitus. Second, only published studies in English were included. Lastly, in addition to genetic factors, various risk factors that can affect the plasma lipid concentration (gender, body weight, ethnicity, environment, etc.) were not considered.

## 5. Conclusions

This meta-analysis demonstrates that *ABCA1* gene polymorphisms (69C>T, 825V>I, and 230R>C) may be associated with lower HDL-C levels. In addition, a significant association was found between the 230R>C polymorphism and lower TC levels. To the best of our knowledge, this is the first meta-analysis to show an association between the *ABCA1* 69C>T, 825V>I, and 230R>C polymorphisms and the plasma lipid concentration. Further studies with a large population and analysis of related risk factors are needed to confirm our findings.

## Figures and Tables

**Table 1:** Characteristics of *ABCA1* 69C>T, 825V>I, and 230R>C studies included in the systematic review.

First Author, Year	Country	Disease	Study Design	Participants (Female %)	Age (Years) (Mean ± SD)	BMI (kg/m^2^) (Mean ± SD)	Genotyping	Studied Lipid	NOS
69C>T									
Hodoğlugil, 2005	Turkey	-	Cohort study	2700 (42.6)	41.0 ± 12.9	25.9 ± 4.5	RFLP, ASO	HDL-C	7
Porchay, 2006	France	IRS	Cohort study	5040 (51.0)	46.8 ± 10.0	24.7 ± 3.8	PCR	HDL-C	6
Abellán, 2010	Spain	-	Cohort study	1367 (50.7)	52.7 ± 18.9	26.3 ± 4.2	Oligo-ligation assay/PCR technology	HDL-C, LDL-C, TC	7
Versmissen, 2011	Netherlands	FH	Cohort study	1686 (53.1)	39.1 ± 12.8	25.0 ± 3.5	PCR, immobilized probe assay	HDL-C, LDL-C, TG, TC	7
Ergen, 2012	Turkey	T2DM, healthy	Case–control study	157 (56.1)	54.7 ± 12.2	27.1 ± 4.9	PCR-RFLP	HDL-C, LDL-C, TG, TC	7
Alharbi, 2013	Saudi Arabia	T2DM, healthy	Case–control study	756 (43.5)	48.3 ± 9.1	29.3 ± 5.7	PCR-RFLP	HDL-C, LDL-C, TG, TC	6
Haghvirdizadeh, 2015	Malaysia	T2DM, healthy	Case–control study	329 (42.2)	58.5 ± 10.7	27.5 ± 5.7	PCR-HRM	HDL-C, LDL-C, TG, TC	5
Li, 2018	China	T2DM, healthy	Case–control study	1122 (42.0)	55.2 ± 11.7	25.7 ± 4.7	MALDI-TOF MS	HDL-C, LDL-C, TC	7
Du, 2020	China	T2DM	Case–control study	996 (50.2)	60.2 ± 8.6	26.4 ± 3.2	PCR, SNaPshot	HDL-C, LDL-C	7
Ghafar, 2020	Egypt	T2DM	Case–control study	104 (62.5)	49.7 ± 9.0	29.2 ± 4.1	TaqMan real-time PCR	HDL-C, LDL-C, TG, TC	7
Yan, 2020	China	T2DM	Case–control study	1086 (51.7)	58.8 ± 9.7	26.8 ± 3.5	SNaPshot	HDL-C, LDL-C	7
825V>I									
Saleheen, 2007	Pakistan	-	Cohort study	200 (36.0)	49.4 ± 5.0	NA	PCR-RFLP	HDL-C	6
Wang, 2010	China	ACI, LI, healthy	Case–control study	476 (34.0)	65.6 ± 10.5	24.1 ± 2.6	PCR-RFLP	HDL-C, LDL-C, TG, TC	7
Cao, 2011	China	-	Cohort study	1323 (52.1)	40.5 ± 16.2	22.2 ± 2.8	PCR-RFLP	HDL-C, LDL-C, TG, TC	7
Kolovou, 2015	Greece	-	Cohort study	432 (80.0)	29.8 ± 6.5	26.0 ± 4.7	PCR-RFLP	HDL-C, LDL-C, TG, TC	6
Dakhil, 2019	Iraq	T2DM, healthy	Case–control study	150 (52.0)	53.4 ± 6.4	30.8 ± 5.9	PCR-RFLP	HDL-C, LDL-C, TG, TC	6
230R>C									
Villarreal-Molina, 2007	Mexico	-	Cohort study	429 (64)	40.1 ± 12.4	27.5 ± 5.5	TaqMan assay	HDL-C, TG, TC	7
Romero-Hidalgo, 2011	Mexico	-	Cross-sectional study	3591 (67.7)	46.7 ± 13.1	28.4 ± 4.9	TaqMan assay	HDL-C, LDL-C, TC	7
Haghvirdizadeh, 2015	Malaysia	T2DM, healthy	Case–control study	329(42.2)	58.5 ± 10.7	27.5 ± 5.7	PCR-HRM	HDL-C, LDL-C, TG, TC	5
Du, 2020	China	T2DM	Case–control study	996 (50.2)	60.2 ± 8.6	26.4 ± 3.2	PCR, SNaPshot	HDL-C, LDL-C	7

SD: standard deviation, BMI: body mass index, NOS: Newcastle–Ottawa scale, IRS: insulin resistance syndrome, FH: familial hypercholesterolemia, T2DM: type 2 diabetes mellitus, ACI: atherothrombotic cerebral infarction, LI: lacunar infarction, NA: not available, RFLP: restriction fragment length polymorphism, ASO: allele-specific oligonucleotide hybridization, PCR: polymerase chain reaction, HRM: high-resolution melt, MALDI-TOF MS: matrix-assisted laser desorption time-of-flight mass spectrometry, HDL-C: high-density lipoprotein cholesterol, LDL-C: low-density lipoprotein cholesterol, TG: triglycerides, TC: total cholesterol.

## Data Availability

No new data were created in this study. Data sharing is not applicable to this article.

## Supplementary Materials

The following are available online at https://www.mdpi.com/article/10.3390/jpm11090883/s1, Figure S1: Funnel plots demonstrating the association between *ABCA1* 69C>T polymorphisms and lipid levels (mmol/L). (**A**) High-density lipoprotein cholesterol; (**B**) low-density lipoprotein cholesterol; (**C**) triglycerides; (**D**) total cholesterol, Figure S2: Funnel plots demonstrating the association between *ABCA1* 825V>I polymorphisms and lipid levels (mmol/L). (**A**) High-density lipoprotein cholesterol; (**B**) low-density lipoprotein cholesterol; (**C**) triglycerides; (**D**) total cholesterol, Figure S3: Funnel plots demonstrating the association between *ABCA1* 230R>C polymorphisms and lipid levels (mmol/L). (**A**) High-density lipoprotein cholesterol; (**B**) low-density lipoprotein cholesterol; (**C**) triglycerides; (**D**) total cholesterol.
