# Supplementary material for: Association between ABCA1 Gene Polymorphisms and Plasma Lipid Concentration: A Systematic Review and Meta-Analysis
Source: J Pers Med. 2021 Sep 3;11(9):883. doi: 10.3390/jpm11090883 (PMC8466567; doi:10.3390/jpm11090883)
Supplement: Supplementary file 1 [file jpm-11-00883-s001.zip › jpm-1333458-supplementary.pptx]

## Slide 1
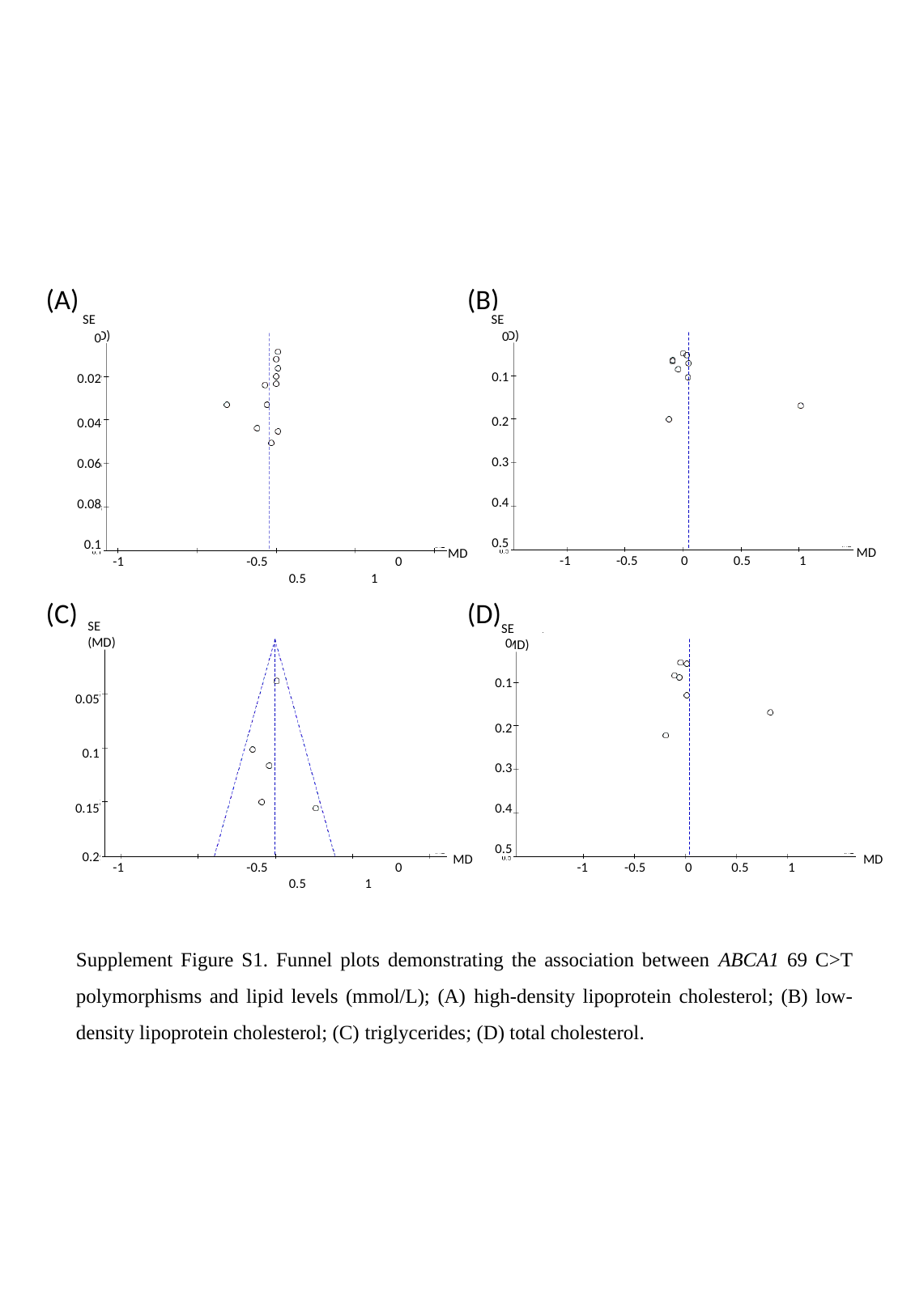

(A)
(B)
SE (MD)
0
0.1
0.2
0.3
0.4
0.5
MD
-1 -0.5	0 0.5 1
SE (MD)
0
0.02
0.04
0.06
0.08
0.1
MD
-1	 -0.5	 0	 0.5	 1
(C)
(D)
SE (MD)
0
0.05
0.1
0.15
0.2
MD
-1	 -0.5	 0	 0.5	 1
SE (MD)
0
0.1
0.2
0.3
0.4
0.5
MD
-1 -0.5 0 0.5 1
Supplement Figure S1. Funnel plots demonstrating the association between ABCA1 69 C>T polymorphisms and lipid levels (mmol/L); (A) high-density lipoprotein cholesterol; (B) low-density lipoprotein cholesterol; (C) triglycerides; (D) total cholesterol.

## Slide 2
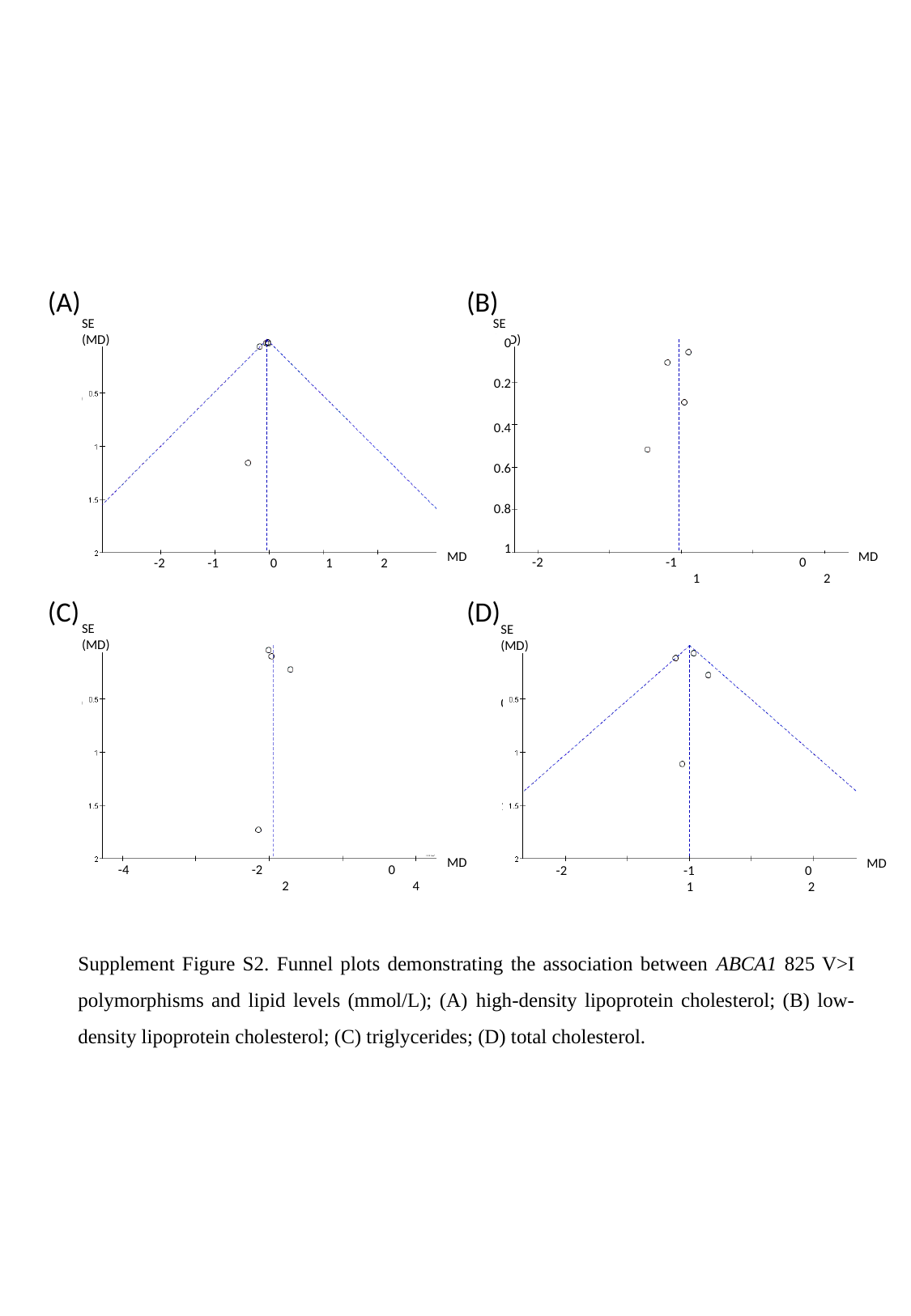

(A)
(B)
SE (MD)
MD
-2 -1 0 1 2
SE (MD)
0
0.2
0.4
0.6
0.8
1
MD
-2	 -1	 0	 1	 2
0
0.5
1
1.5
2
(C)
(D)
SE (MD)
MD
-4	 -2	 0	 2	 4
SE (MD)
MD
-2	 -1	 0	 1	 2
0
0.5
1
1.5
2
0
0.5
1
1.5
2
Supplement Figure S2. Funnel plots demonstrating the association between ABCA1 825 V>I polymorphisms and lipid levels (mmol/L); (A) high-density lipoprotein cholesterol; (B) low-density lipoprotein cholesterol; (C) triglycerides; (D) total cholesterol.

## Slide 3
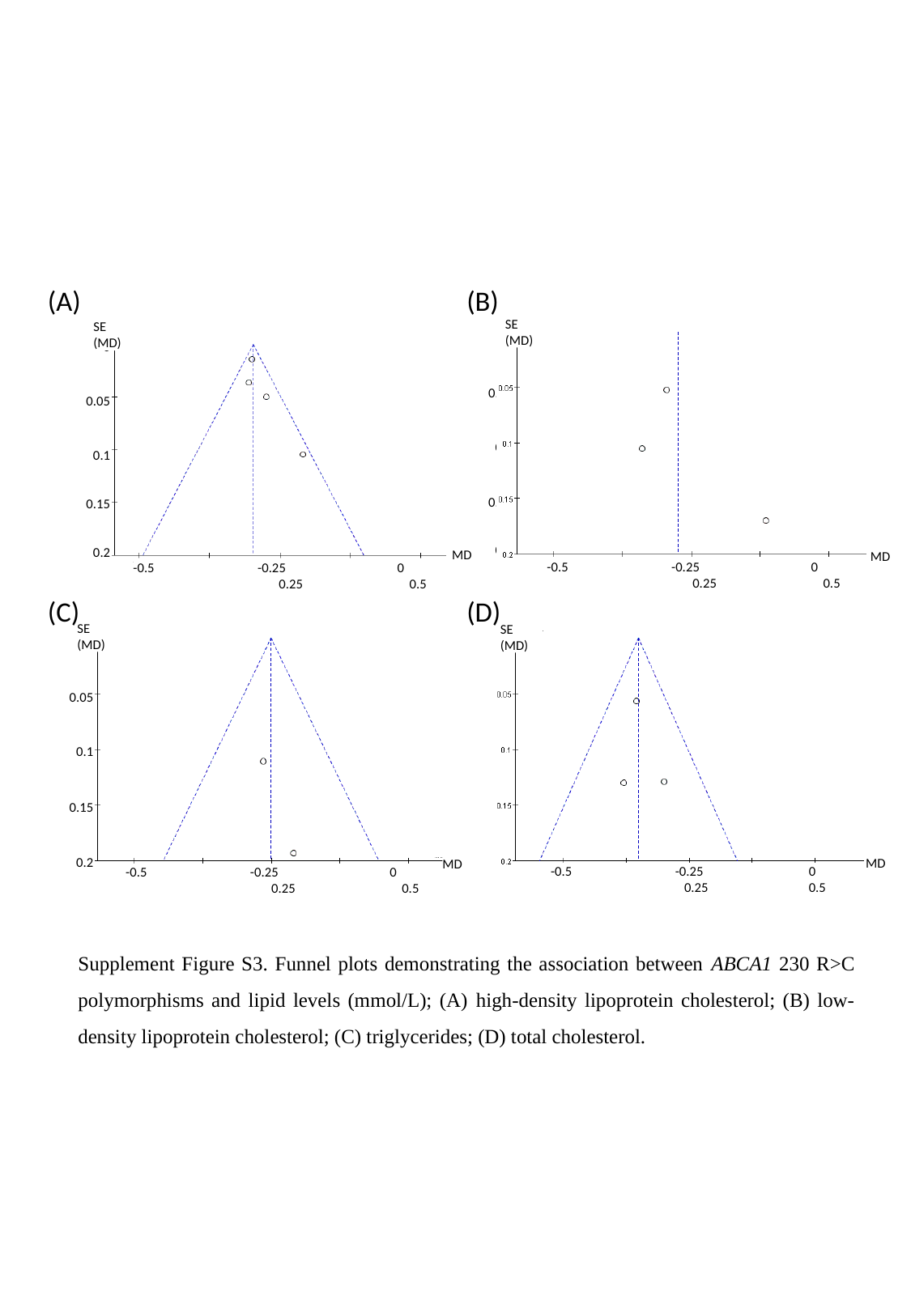

(A)
(B)
SE (MD)
MD
-0.5	 -0.25	 0	 0.25	 0.5
SE (MD)
0
0.05
0.1
0.15
0.2
MD
-0.5	 -0.25	 0	 0.25	 0.5
0
0.05
0.1
0.15
0.2
(C)
(D)
SE (MD)
0
0.05
0.1
0.15
0.2
MD
-0.5	 -0.25	 0	 0.25	 0.5
SE (MD)
MD
-0.5	 -0.25	 0	 0.25	 0.5
Supplement Figure S3. Funnel plots demonstrating the association between ABCA1 230 R>C polymorphisms and lipid levels (mmol/L); (A) high-density lipoprotein cholesterol; (B) low-density lipoprotein cholesterol; (C) triglycerides; (D) total cholesterol.
